# Tuberculosis among children, adolescents and young adults in the Philippines: a surveillance report

**DOI:** 10.5365/wpsar.2017.8.4.011

**Published:** 2018-11-09

**Authors:** K Snow, R Yadav, J Denholm, S Sawyer, S Graham

**Affiliations:** a Centre for International Child Health, University of Melbourne, Department of Paediatrics and Murdoch Children’s Research Institute, Royal Children’s Hospital, Melbourne, Australia.; b School of Population and Global Health, University of Melbourne, Melbourne, Australia.; c WHO Country Office for the Philippines, Manila, Philippines.; d Victorian Tuberculosis Program, Melbourne Health, Melbourne, Australia.; e Department of Microbiology and Immunology, University of Melbourne, Melbourne, Australia.; f Centre for Adolescent Health, Royal Children’s Hospital, Melbourne, Australia.; g Murdoch Children’s Research Institute, Melbourne, Australia.; h Department of Paediatrics, The University of Melbourne, Melbourne, Australia.; i International Union Against Tuberculosis and Lung Disease, Paris, France.

## Abstract

The Philippines, a country with a young population, is currently experiencing an intense and persistent tuberculosis epidemic. We analysed patient-based national surveillance data to investigate the epidemiology of reported tuberculosis among children (aged 0–9 years), adolescents (aged 10–19 years) and young adults (aged 20–24 years) to better understand the burden of disease and treatment outcomes in these age groups.

Descriptive analyses were performed to assess age-related patterns in notifications and treatment outcomes. Data quality was assessed against international benchmarks at the national and regional levels.

Overall, 27.3% of tuberculosis notifications for the Philippines in 2015 pertained to children, adolescents and young adults aged 0–24 years. Treatment outcomes were generally favourable, with 81% of patients being cured or completing treatment. The data quality assessment revealed substantial regional variation in some indicators and suggested potential underdetection of tuberculosis in children aged 0–4 years.

Children, adolescents and young adults in the Philippines constitute a substantial proportion of patients in the national tuberculosis surveillance data set. Long-term progress against tuberculosis in the Philippines relies on improving the control of tuberculosis in these key age groups.

## Introduction

The World Health Organization (WHO) estimates that 1.8 million people living in the Western Pacific Region developed active tuberculosis (TB) in 2016, and of these, 573 000 (32%) lived in the Philippines. (*1*) The Philippines has one of the highest TB incidence rates in the Region, estimated at 554 cases per 100 000 in 2016, a rate that has not declined significantly since 2007. (*1*)

Half (52%) of the population of the Philippines is under 25 years of age, compared to a regional average of 43%. (*2*) Age influences TB risk in a variety of biological and social ways, and TB epidemiology changes with population age structure as nations undergo demographic shifts. (*3*) Children under 5 years of age are at high risk of developing clinical TB after infection and are prone to developing severe forms of TB such as TB meningitis and disseminated TB, particularly if not protected by Bacillus Calmette-Guérin (BCG) vaccination. (*4*) In contrast, adolescents and young adults (“young people,” aged 10–24 years) more often develop infectious pulmonary TB. (*4*) Young people who attend educational institutions or reside in institutional settings may have multiple extended respiratory contacts per day. (*5*) Furthermore, recent research suggests that young people may be at increased risk of discontinuing TB treatment before completion (previously referred to as “defaulting”). (*6*)

The first step in improved TB control is to understand the epidemiology of the disease to inform and implement evidence-based interventions for at-risk groups. The aim of this study was to describe the age-related epidemiology and outcomes of reported TB in the Philippines using patient-based national TB surveillance data with a focus on individuals aged 0–24 years. The second aim was to evaluate surveillance data quality against international benchmarks for childhood TB.

## Methods

This analysis used data from the national TB surveillance system of the Philippines, the Integrated Tuberculosis Information System (ITIS). (*7*) This is a case-based electronic surveillance system with data entry performed at the health-facility level. We calculated crude notification rates nationally and for each of the Philippines’ 17 regions for the 2015 calendar year. We also calculated age- and sex-specific rates using population estimates from the 2015 census. (*8*) We calculated the proportion of TB that was extra pulmonary by age group, and we assessed the risk of each unfavourable treatment outcome by age group and sex (using all registered patients of each group as the denominator).

For consistency with available population data, we analysed five-year age groups across the 0–24-year age span (young children aged 0–4, older children aged 5–9, young adolescents aged 10–14, older adolescents aged 15–19 and young adults aged 20–24) to allow detailed description of outcomes across different age groups. To compare the risks of unfavourable treatment outcomes (premature discontinuation, treatment failure, death or no recorded outcome) by age, we stratified the adult age group into 25–49 and ≥ 50 years as mortality on TB treatment is known to be higher among older adults. (*3*) We calculated risk ratios with 95% confidence intervals for successful treatment using the 25–49 year age group as the reference group.

To assess the quality of the surveillance data regarding child TB at national and regional levels, we evaluated data in ITIS using selected items from WHO’s Standards and Benchmarks Checklist for TB surveillance data. We evaluated data quality using the two WHO benchmarks for TB surveillance relevant to childhood TB: (*1*) in a middle-income country, 5–15% of all new TB patients are expected to be younger than 15 years; (*2*) and the ratio of children 0–4 years old to those aged 5–14 years old is expected to be between 1.5:1 and 3.0:1. (*9*) We repeated this assessment by administrative region to determine the variability in these key indicators within the country. We also calculated the percentages of notifications by five-year age groups to compare regional variation in age-related burden of reported disease.

Unit record data were managed and analysed in Stata 13 (Statacorp, College Station, Texas, USA), and aggregate data were managed and analysed in Microsoft Excel (Microsoft, Redmond, Washington, USA). Patients with multidrug-resistant TB were excluded from all analyses.

### Ethics

This study was approved by the Human Ethics Advisory Group at the Department of Paediatrics, University of Melbourne and by the National Tuberculosis Program of the Philippines, who provided access to the data.

## Results

### Epidemiology

There were 299 005 patients of known-age and sex registered on treatment for new or relapsed TB in the Philippines in 2015. All records in the data set were complete with regard to age, sex, registration date, treatment history and a patient identifier. A very small number (0.4%) of records did not specify the basis for diagnosis (clinical versus microbiologic). The crude notification rate for the country was 296 cases per 100 000 person-years. At the regional level, notification rates varied from 144 cases per 100 000 person-years to 364 cases per 100 000 person-years (median rate = 291, interquartile range [IQR]: 262–333). Nationwide, 38 694 (12.8%) of the patients were children and young adolescents aged 0–14 years; 43 923 (14.5%) were older adolescents and young adults aged 15–24 years. Only 1.9% of all reported TB cases were classified as exclusively extrapulmonary in nature.

**Fig. 1:**
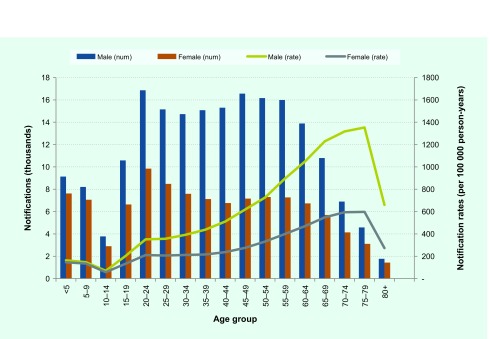
Notifications (thousands) and notification rates for TB by age and sex (thousands of cases per 100 000 person years), the Philippines, 2015

The frequency and rates of new or relapsed TB, stratified by age group and sex, are shown in **Fig. 1**. The number of notifications and the notification rates were higher in males than females in most age groups. The 20–24 year age group had the highest absolute number of notifications nationally in both sexes; however, per capita the rates of notification rose steadily across the adult age groups, peaking in the 75–79 year age group and then falling in the ≥ 80 year age group.

Of all new and relapsed patients registered in 2015, 242 629 (81.2%) were treated successfully according to their final recorded outcomes, 36 881 (12.3%) had no outcome recorded, and the remaining 19 495 (6.5%) experienced an unfavourable outcome (discontinuation, treatment failure or death). Young children (aged 0–4 years) were more likely to be treated successfully than adults aged 25–49 (risk ratio [RR] = 1.06, 95% confidence interval [CI]: 1.05, 1.07). Compared to adults aged 25–49 years, young adults (aged 20–24 years) had the same likelihood of treatment success (RR = 1.01, 95% CI: 1.00–1.02), while adults over 50 years of age were slightly less likely to be treated successfully (RR = 0.97, 95% CI: 0.96–0.97). Missing outcome data were equally common in all age groups, varying between 11.2% in children under 5 and 13.0% in adolescents aged 10–14 years.

**Fig. 2:**
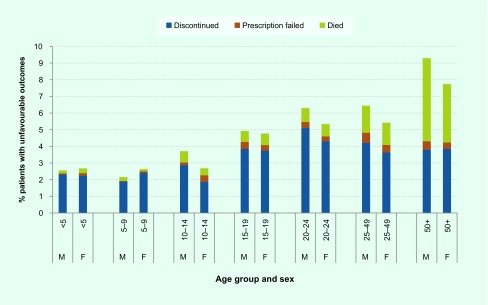
Percentage of patients with unfavourable treatment outcomes by age and sex, new and relapse TB patients, the Philippines, 2015

Treatment discontinuation was somewhat more common among adolescents and adults than among children. Young men aged 20–24 years had the highest risk of discontinuation of any age group: 5.1% of young men discontinued treatment prematurely (**Fig. 2**).

### Data quality indicators

The proportion of new and relapsed TB among children and young adolescents aged 0–14 years was 12.8% nationally, but varied substantially by region, ranging from 3.5% to 27.9% of all TB cases, suggesting significant geographic variation in data quality regarding paediatric TB. The ratio of children under 5 to children and young adolescents aged 5–14 years ranged from 0.3:1 to 1.1:1 across the regions, with no region meeting the WHO benchmark of 1.5:1. Nationally, the ratio was 0.8:1.

## Discussion

This study reviewed the epidemiology and outcomes of TB in the Philippines in 2015 using the patient-based national surveillance data set. (*7*) We observed a high proportion of notifications among children, adolescents and young adults albeit with regional variation in data quality indicators for childhood TB. Treatment outcomes were largely favourable, although outcome data were missing for a substantial proportion of patients across all age groups.

Consistent with the country’s young population, over one quarter of TB patients in the Philippines are children, adolescents and young adults. This proportion is comparable to that seen in Cambodia (26%) but markedly higher than that in Viet Nam (9.6%) or the Lao People's Democratic Republic (9.5%), the other high-burden countries in the Western Pacific Region. (*10*) The high proportion of patients aged under 25 in the Philippines has implications both in the short-term, during which the burden of disease among children and young people is substantial, and in the longer term, as the current generation ages with a high prevalence of latent TB infection. Improving the quality of TB prevention, diagnosis and management among these age groups would contribute to TB control in the Philippines both immediately and in the longer term.

We identified potential problems in surveillance data quality in some regions that warrant further investigation. The proportion of cases affecting children has risen considerably in recent years from 2.1% in 2013 (*10*) to 12.8% in 2016, following national efforts to improve detection of childhood TB. In a high transmission setting, children aged 0–4 years are disproportionately impacted due to their high risk of progression to active disease relative to older children. (*4*) That the number of notifications from 0–4 year olds and 5–14 year olds is almost equal in the Philippines (rather than the expected ratio of 1.5–3.0:1) (*9*) suggests that TB in children aged 0–4 is being underdiagnosed or underreported.

The regional variability in the proportion of reported TB cases contributed by children and young adolescents aged 0–14 suggests substantial variation in diagnostic and reporting practices within the country. The low mortality rate among young children likewise suggests the possibility of underdiagnosis or underreporting of disseminated TB and TB meningitis.

The proportion of notifications in the 10–14 year age group was consistently low throughout the Philippines in spite of marked variations in other indicators between the regions. The transiently reduced risk of TB in this age group has been well described in historical TB epidemics, (*11*) though there may also be an elevated risk of underdetection in this age group, who can fall between child and adult health services. (*12*) Notification rates in the Philippines rise markedly across the adolescent and young adult age groups as seen in both historical and modern TB epidemics in many settings. (*3*, *4*)

The most recent national TB prevalence survey in the Philippines revealed substantial gaps in case detection in the country with a prevalence to notification ratio of 3.0. (*13*) The survey observed the greatest gap between notifications and prevalence in the 15–24 year age group (the youngest group included in the survey), suggesting that the true burden of disease in this age group is over fourfold higher than documented by the notification data presented here. (*13*)

The major limitations of this study reflect the limitations of the data source used. We used data from the first year in which ITIS was operational nationally, and some facilities and regions may still have been adjusting to the new system. TB surveillance systems capture a limited number of variables, and some details that would have been valuable were not available, for example, dates of diagnosis and subsequent disengagement from care or BCG vaccination status. If age, diagnostic data or treatment outcome were recorded inaccurately, this will have affected our results. Nonetheless, the data used in this study are the most detailed and complete source of data on TB in the Philippines, and a data quality evaluation was included in this analysis.

This study described the epidemiology and outcomes of TB among children and young people in the Philippines who constitute one quarter of all registered patients nationally. Our study together with the recent national prevalence survey (*13*) highlight the need for improved TB control in the younger age groups who will play a key role in the Philippines’ progress against TB over the coming decades.
